# Efficacy and safety of oral traditional Chinese patent medicine in treatment of liver stagnation and spleen deficiency of depression: A protocol for systematic review

**DOI:** 10.1097/MD.0000000000019142

**Published:** 2020-02-14

**Authors:** Ying Yu, Gong Zhang, Tao Han, Hailiang Huang

**Affiliations:** a 2018 Level Doctor's Degree Graduate Class; b 2017 Level Doctor's Degree Graduate Class, College of Traditional Chinese Medicine, Shandong University of Traditional Chinese Medicine; c Graduate Office of Shandong University of Traditional Chinese Medicine; d College of Rehabilitation Medicine, Shandong University of Traditional Chinese Medicine, Jinan, China.

**Keywords:** Chinese patent medicine, depression, liver stagnation and spleen deficiency, network meta analysis, protocol

## Abstract

**Background:**

Depression is a kind of chronic and recurrent mental disorder, the main clinical characteristics of the patients are marked and persistent depression. At the same time, it is often accompanied by chronic physical disease, cognitive impairment, and functional damage, which is one of the common diseases that seriously threaten human health. At present, 3 kinds of oral Chinese patent medicine have clinical comparability in the treatment of depression of liver stagnation and spleen deficiency, but there is no evidence for clinical efficacy and safety. Therefore, this study aims to integrate the clinical related syndromes of direct and indirect comparison by using systematic evaluation and network meta-analysis (NMA). According to the data, the different Chinese patent medicines with the same evidence body for the treatment of the disease are collected, analyzed, and sequenced in a quantitative and comprehensive way, and then the advantages and disadvantages of the efficacy and safety between different Chinese patent medicines are screened out to get the best choice scheme, thus providing reference value and evidence-based theoretical evidence for the clinical optimization of drug selection.

**Methods:**

Comprehensive retrieval of China National Knowledge Infrastructure, Chinese scientific journal database (VIP), China biological feature database (CBM) and WANFANG Data Chinese electronic database and the Cochrane Library, PubMed, Web of Science, and EMBASE foreign database. Search and publish the clinical randomized controlled trials of these 3 Chinese patent medicines combined with fluoxetine compared with fluoxetine. The retrieval time is from the establishment of the database to October 31, 2019. The 2 first authors will screen the literatures that meet the inclusion criteria, extract the data independently according to the predesigned rules, and evaluate the literature quality and bias risk of the included research according to the Cochrane 5.1 manual standard. R and Aggregate Data Drug Information System software were used for data consolidation and NMA to evaluate the ranking probability of all interventions.

**Results:**

This result will show that the best oral Chinese patent medicine to assist the treatment of liver stagnation and spleen deficiency depression provides reliable evidence.

**Conclusion:**

This study will provide systematic evidence-based medicine evidence for TCM assisted treatment of depression of liver stagnation and spleen deficiency type, and help clinicians, patients with depression and decision-makers to make more effective, safer, and economic optimal treatment plan in the decision-making process.

**PROSPERO registration number:**

CRD42019115695.

## 1 Introduction

Depression is one of the serious mental disorders with high morbidity, high disability, high recurrence rate, and high suicide rate. The main clinical characteristics of patients are significant and persistent depression, which is often accompanied by chronic physical disease, cognitive impairment, and functional impairment. According to the statistics of the World Health Organization's global disease burden cooperation research, there are 3.22 million people suffering from depression, accounting for 4.4% of the global population, 50% to 80% of the patients have experienced relapse, and >50% of the patients with depression have committed suicide,^[1–4]^ which is one of the common diseases that seriously threaten human health. At the same time, mental disorders and coronary heart disease have been regarded as 2 major global diseases. With the rapid development of modern society and economy, the acceleration of life rhythm and the increase of work competition pressure, the incidence of the disease shows the trend of increasing year by year and the tendency of youth, which not only seriously puzzles the normal life and work of patients, but also brings heavy burden to the family and society. Therefore, it is urgent to effectively improve the clinical cure rate of the disease, shorten the course of disease, and reduce the side effects as the focus of research. At present, western medicine mostly uses antidepressant chemicals such as monoamine oxidase inhibitor, selective 5-hydroxytryptamine (5-HT) or noradrenaline reuptake inhibitor, 3/4 ring antidepressants, and so on. Although they have short-term clinical efficacy, along with the long-term medication cycle prolongation, the patients will have different degrees of adverse reactions, so it seriously restricts the patients’ treatment compliance and the curative effect after the disease.^[5]^

Traditional Chinese medicine (TCM), as an important part of complementary and alternative medicine, is good at using the method of combination of holistic view and syndrome differentiation view. It has unique clinical advantages in the treatment of the disease. In addition, 3 kinds of oral Chinese patent medicines are processed from natural herbal medicine, which can not only greatly shorten the healing time of clinical treatment, but also the clinical convenience and standardization of oral Chinese patent medicine, safety, taste optimization, and other features can greatly improve the subjective initiative and treatment compliance of clinical patients, which has been widely recognized and applied in clinical practice in China. Therefore, we are first classifies and extracts the syndrome elements through the expert experience syndrome differentiation, summarizes the distribution characteristics of common syndrome elements of depression, and finds that the type of liver stagnation and spleen deficiency is the most common clinical syndrome of depression patients. At the same time, data mining and statistical analysis are carried out on the literature of using Chinese patent medicine to treat this syndrome type, and the ranking of clinical auxiliary treatment of liver stagnation and spleen deficiency depression is selected. The top 3 commonly used Chinese patent medicines are Shugan Jieyu capsule, Jieyu pill, and Xiaoyao pill, the control group is fluoxetine, which is currently the first choice in clinical use at home and abroad, and is also a new generation of classic antidepressants recommended in the International Classification of Diseases (ICD-10), American Psychiatric diagnosis and clinical guidelines.^[6]^ Three kinds of Chinese patent medicine combined with fluoxetine are compared with fluoxetine in clinical practice. Therefore, in the case of relatively consistent syndrome type, dosage form and control type of Chinese patent medicine, the 3 kinds of Chinese patent medicine auxiliary treatment have clinical comparability. However, most of the current studies only report the efficacy of oral Chinese patent medicine in the treatment of depression of liver stagnation and spleen deficiency type by conventional paired meta-analysis, but there is no evidence-based evaluation of the clinical efficacy and safety of the 3 Chinese patent medicine in the treatment of depression of liver stagnation and spleen deficiency type. Therefore, the purpose of this study is to use the network meta-analysis (NMA) method to integrate the clinical relevant evidence of direct and indirect comparative relationship, and draw this conclusion. The clinical effective rate, Hamilton Depression Scale (HAMD) score and Treatment Emergent Symptom Scale (TESS) score of 3 kinds of Chinese patent medicine adjuvant treatment were analyzed by quantitative comprehensive statistics, and then according to the advantages and disadvantages of the index efficacy, the probability ranking was carried out, and then the best clinical treatment scheme was selected, which provided reference value and evidence-based medicine evidence for clinical optimization of drug selection.^[7]^

## 2 Materials and methods

### 2.1 Protocol and registration

We will complete this protocol for systematic evaluation and NMA, which follows the statements of “Cochrane manual for systematic evaluation of interventions” and “protocol for systematic evaluation and network meta-analysis” (PRISMA-P) in accordance with recognized standards. A report on the further results of this study will be submitted in accordance with the guidelines of the PRISMA NMA extension statement.^[8]^ In July 2019, we have obtained the registration number (CRD42019115695) of this study on the platform of PROSPERO (https://www.crd.york.ac.uk/prospero/). Because the NMA protocol has been approved by the local agency review committee and the ethics committee, it does not involve privacy information and does not require further ethical approval and informed consent.

### 2.2 Information sources

By using the computer retrieval technology, the clinical randomized controlled trials (RCTs) of 3 kinds of Chinese patent medicine assisted treatment of depression of liver stagnation and spleen deficiency type were searched. The primary search was selected and the set period was from the establishment of the database to October 31, 2019. The computer retrieval electronic database included China National Knowledge Infrastructure, China biological feature database (CBM), WANFANG data, Chinese scientific journal database (VIP) and other Chinese databases, as well as the Cochrane Library, PubMed, Web of Science and EMBASE, and other foreign databases. Chinese search terms include depression, depression, Shugan Jieyu capsule, Jieyu pill, Xiaoyao Pill, fluoxetine, random, etc. English search terms include (Depressions OR Depressive Symptoms OR Emotional Depres∗) and (Shugan Jieyu capsule OR Jieyu Pill OR xiaoyao Pill OR Fluoxetine and (Random∗OR randomized controlled trials OR clinical randomized controlled trials).

When searching the literature, the subject words and free words shall be searched separately, and the relevant free words and terms shall be used for comprehensive search. Meanwhile, the research of WHO international clinical trial registration platform and ClinicalTrials.gov shall be searched to determine the additional potential trial registration. In addition, the relevant journals shall be searched in the reference literature, and the relevant literature shall be tracked, and Google scholars shall be used together Baidu academic and other relevant search engines conduct relevant research on the Internet by hand, and will provide data for all relevant authors and major researchers to supplement the incomplete report or unpublished research of the original paper. We will try our best to ensure that the primary search work is comprehensive so as not to lose valuable research materials. At the same time, according to the Participant-Intervention-Comparator-Outcomes-Study design (PICOS) search principle, we will include the research that meets the standards and organize and create the database. In Table 1, the preliminary search strategy of PubMed database is taken as an example to summarize the preliminary search strategy, which will be adjusted according to the requirements of other electronic databases related to keywords.

**Table 1:**
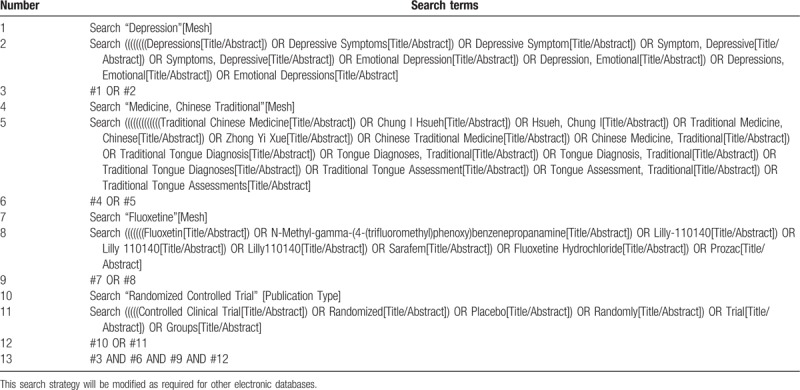
Search strategy used in PubMed database.

### 2.3 Eligibility criteria

The design of inclusion criteria and exclusion criteria in this study is based on the 5 main principles of PICOS.

#### 2.3.1 Type of participant

The patients were single depression of liver stagnation and spleen deficiency type, and their age, sex, and race were unlimited. The diagnostic criteria of depression used should conform to one of the following: the third edition of Chinese classification and diagnostic criteria for mental disorders, ICD-10, the American Manual of diagnosis and statistics for mental disorders (*Diagnostic and Statistical Manual of Mental Disorders, Fifth Edition*), and the diagnostic criteria of syndrome types of TCM should conform to the diagnostic and treatment guidelines of the diagnostic efficacy criteria of TCM syndrome types or the syndrome differentiation criteria of TCM.

#### 2.3.2 Type of interventions and comparators

In the treatment group, Shugan Jieyu capsule or Jieyu pill or Xiaoyao Pill were used when the diagnostic criteria, efficacy criteria, and basic treatment were consistent. These interventions can be used alone or in combination with fluoxetine. In the control group, only western medicine fluoxetine (recommended by internationally recognized clinical guidelines) was used.

#### 2.3.3 Type of outcomes

The predetermined evaluation results mainly include clinical efficacy, HAMD score, TESS, and other details.

#### 2.3.4 Type of study

The literature included were RCTs with no limitation on language and blind or assignment concealment. When the clustering effect is considered, the cluster RCT will be included. Although more and more clinical trial reports are coming from mainland China, people are concerned about the quality of these studies, so it is specially explained that as long as the Chinese trial is approved by the local institutional review committee and registered in the international database, we will include its research into the scope of the study. In addition, the author will exclude the non-RCT, case report, experience summary, self-control and review literature, animal experimental research, and repeatedly published literature. At the same time, the diagnosis of depression is not clear or literature combined with other diseases, the efficacy judgment standard of the test group and the control group is not clear, and the treatment measures involve other treatment and affect the final treatment of causality judgment literature, literature with unclear research results, incomplete data or no connection with the full text author is also excluded.

### 2.4 Study selection and data extraction

**Figure 1:**
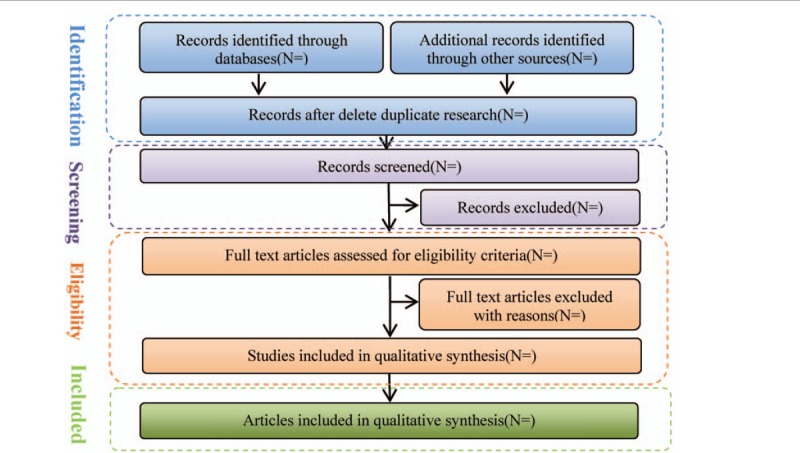
Flow diagram of study.

According to the retrieval strategy of the above electronic databases, 2 researchers searched the electronic databases in Chinese and English, used EndnoteX8 software to search the repeated information, combined the literature retrieval results in different databases, established the information database, and downloaded the full text. Then 2 first authors independently extract the data for preliminary screening, extract the data according to the predetermined table, take cross-check and review, and mark down the reasons for each excluded study, invite the third review researcher to jointly discuss, and make a final decision on the research with different opinions. The process of study selection is summarized in the PRISMA flowchart in Figure 1.^[9,10]^

Data extraction content includes basic information of the included literature (including the first author, published journal and year, research topic). Relevant information of the treatment group and the control group in the literature (including the number of cases, total cases, age, intervention measures, course of treatment, outcome indicators). Design type and quality evaluation information of the included literature.

### 2.5 Study quality evaluation

According to the quality evaluation standard of Cochrane system evaluator manual, RevMan quality evaluation tool was used to evaluate the methodological quality of the included study, including random method, assignment concealment, blind method, outcome data integrity, selective report, number of dropped cases, follow-up, and other biases. Each project was divided into 3 options: high risk, low risk, and uncertainty risk, According to the description of the above aspects in the included research, the 2 first authors independently completed the quality evaluation results of the included literature. If there are differences in the results, it is necessary to invite the third researcher to help each other discuss and make interpretation and quality evaluation. According to the standards of Cochrane manual, literature quality assessment and bias risk assessment were carried out. R software and Aggregate Data Drug Information System (ADDIS) software are used for statistical software, data integration, and NMA.^[11,12]^

Consulting GRADE handbook, the assessment which would be carried out through the Grading of Recommendations Assessment, Development, and Evaluation (GRADE, https://gradepro.org/)^[13,14]^ by 2 independent authors will be designated into 4 grades: high quality, moderate quality, low quality, and very low quality.

### 2.6 Data synthesis and statistical methods

#### 2.6.1 Pairwise and network meta analysis

First of all, the author used RevMan software to analyze the direct comparison results from the literature. Secondly, for the results of indirect comparison, the author will use R and ADDIS software for data merging and NMA.^[15]^ At the same time, the network diagram and anecdotal sequence diagram of various interventions are drawn. R programming language starts NETMETA program, and calls Bayes Markov chain Monte Carlo (MCMC) algorithm to analyze and map the data of random effect model. ADDIS software uses related instructions to call the data results of the random effect model based on Bayesian MCMC algorithm for prior evaluation and processing (4 chains are used for simulation analysis, the initial value is 2.5, the iteration step is refined by 10, the number of iterations is adjusted by 20,000, and the number of simulation iterations is 50,000).^[16,17]^

*P* < .05 and 95% confidence interval (CI) value were used as the standard of statistical difference, odds ratio value was used as the statistical value of efficacy analysis. Weighted mean difference (MD) or standardized MD was used as the measurement data, and 95% CIs were used for each effect. In order to provide indirect comparison of 3 oral Chinese patent medicines, we will make a network diagram. The network diagram is mainly composed of nodes and lines. Among them, the node represents a kind of treatment method, the node connected by a straight line represents a direct or indirect comparison between the 2, and the node size represents the number of objects receiving this treatment. The thickness of this line represents the number of studies. Then, we will analyze the results of all direct or indirect comparisons to evaluate which of the 3 oral Chinese patent medicines is the best treatment for patients with depression of liver stagnation and spleen deficiency type, and estimate the rank probability of each group based on MCMC method.

#### 2.6.2 Assessment of heterogeneity

Heterogeneity will be evaluated by Cochrane. For each pairing comparison, statistical heterogeneity will be evaluated by *I*^2^ index, subgroup analysis based on the heterogeneity factors, and study by χ^2^ test. Evaluate the clinical and method heterogeneity of the included study, and compare the fitting degree of fixed effect model and random effect model. If each study in the subgroup has statistical homogeneity (*P* ≥ .1, *I*^2^ ≤ 50%), the fixed effect mode is used for meta-analysis. Otherwise, the causes of heterogeneity are analyzed first, and the random effect mode is used for meta-analysis without obvious clinical heterogeneity (*P* < .1, *I*^2^ > 50%), and the possible causes of heterogeneity are found out from both clinical and methodological aspects. If the clinical trial data provided cannot be meta analyzed, descriptive analysis shall be conducted.^[18]^

#### 2.6.3 Subgroup and sensitivity analyses

If the result of meta-analysis is positive and there are more than 3 included studies, R software shall be used to conduct sensitivity analysis on the statistical results, and meta-analysis shall be carried out again for each excluded study, and the results shall be compared with those before exclusion. If there is no substantial change in the comparative analysis, the results are stable. Otherwise, the data results are not stable. If significant heterogeneity is found, subgroup analysis will be envisaged based on treatment time, age, race, sex, and quality of study to investigate possible sources of heterogeneity.^[19]^

#### 2.6.4 Assessment of inconsistency

The Node-Split Model of ADDIS software is used to test the inconsistency. If there is no statistical difference (*P* > .05) in each study within the subgroup, it indicates that the heterogeneity of the included study is small, so the consistency model is used for analysis. Otherwise, the Inconsistency Model is used for analysis. Potential Scale Reduced Factor (PSRF) reflects convergence. When PSRF is close to 1 or equal to 1, it indicates that it has achieved better convergence efficiency and the results of consistency model analysis are reliable.^[16]^

#### 2.6.5 Publication bias

If more than 5 studies are included, R software is used to analyze the potential publication bias, and the figure is inverted funnel-shaped and symmetrical, which indicates that the possibility of publication bias is relatively small. If the figures are biased, it indicates that there is a greater possibility of publication bias.

## 3 Discussion

The Shugan Jieyu capsule, Jieyu pill, and Xiaoyao pill selected in this study are 3 kinds of pure Chinese medicine oral preparations for the treatment of depression of liver stagnation and spleen deficiency type. The 3 kinds of Chinese patent medicines are all made from natural antidepressant herbs, which have better functions of soothing the liver and regulating qi, strengthening the spleen, and calming the nerves. The mechanism of action is to exert the synergism of drugs through multicomponent, multitarget, multilevel, and multichannel of TCM, so as to strengthen the inhibition of cerebral cortex and make the excitability of subcortical decreased to play a sedative role, or to promote the excitability of dopamine nerve in hypothalamus and hippocampus, which can effectively inhibit the function of 5-HT nerve, and regulate each of them through the overall compatibility of TCM system to play the best antidepressant effect.^[20]^

In the case of relatively uniform TCM syndrome type and Chinese patent medicine dosage form, the 3 kinds of Chinese patent medicine adjuvant treatment have clinical comparability. But at present, there is no unified standardized standard and treatment principle in the treatment of depression of liver stagnation and spleen deficiency type by TCM, and most of the current studies only report the comparison of the efficacy between the 2 drugs, but there is still a lack of NMA of the clinical efficacy and safety of the 3 Chinese patent medicine in the treatment of depression. Therefore, the purpose of this study is to use a high-quality system to evaluate the commonly used antidepressant Chinese patent medicines, and to use the NMA method to obtain the analysis of the clinical efficiency, HAMD score, and TESS score of the 3 Chinese patent medicine adjuvant treatment, so as to determine the antidepressant effect of the 3 Chinese patent medicines, and then rank the probability according to the advantages and disadvantages of the index effect. Then, the best evidence of clinical treatment measures will be screened out, and the quality of the evidence will be evaluated by the hierarchical method.

NMA also, however, has some limitations, including publication bias, clinical heterogeneity, and selection bias, but we still hope that this study can provide the best possible drug selection and reliable evidence-based medicine for the clinical practice, and to some extent, provide some new insights for TCM in the treatment of depression.

At present, the protocol for the NMA has been registered on the international system review expectation register (CRD42019115695), which will follow the guidelines of “Cochrane Intervention System Review Manual” and “ Prisma-P statement.” In addition, if the protocol needs to be amended, there will be a description of the amendment with the reason and the date.

## Author contributions

**Conceptualization:** Ying Yu, Gong Zhang.

**Data curation:** Ying Yu, Gong Zhang.

**Formal analysis:** Ying Yu, Gong Zhang.

**Funding acquisition:** Ying Yu, Tao Han, Hailiang Huang.

**Investigation:** Gong Zhang, Tao Han, Hailiang Huang.

**Methodology:** Ying Yu, Gong Zhang.

**Project administration:** Ying Yu, Tao Han, Hailiang Huang.

**Resources:** Ying Yu, Gong Zhang.

**Software:** Ying Yu, Gong Zhang.

**Supervision:** Tao Han, Hailiang Huang, Gong Zhang.

**Validation:** Gong Zhang, Tao Han, Hailiang Huang.

**Visualization:** Ying Yu, Gong Zhang.

**Writing-original draft:** Ying Yu, Gong Zhang.

**Writing – review and editing:** Ying Yu, Gong Zhang.

Ying Yu orcid: 0000-0002-3404-0006

Gong Zhang orcid: 0000-0002-9425-4727

Tao Han orcid: 0000-0003-3463-3319

Hailiang Huang orcid: 0000-0003-1186-4501

Ying Yu orcid: 0000-0002-3404-0006.
